# Reconsidering blood refusal: Implications of updated Jehovah’s Witness acceptance of autologous transfusion

**DOI:** 10.46989/001c.172919

**Published:** 2026-10-29

**Authors:** Raeshun T. Glover, Brian D. Adkins, Garrett S. Booth, Bipin N Savani, Jeremy W. Jacobs

**Affiliations:** 1 Department of Pathology, University Hospitals Cleveland Medical Center, Case Western Reserve University, Cleveland, OH 44106, USA; 2 Department of Pathology University Hospitals Cleveland Medical Center; 3 Department of Pathology, University of Texas Southwestern Medical Center, Dallas, USA; 4 Children’s Health, Dallas, Texas, USA; 5 Department of Pathology, Microbiology and Immunology, Vanderbilt University, Nashville, Tennessee, USA; 6 Division of Hematology-Oncology, Department of Medicine, Vanderbilt University Medical Center, Nashville, TN, USA

**Keywords:** Jehovah’s Witness, Blood refusal, Autologous transfusion, Bloodless medicine

## Abstract

Refusal of blood transfusion has historically been associated with members of the Jehovah’s Witness (JW) faith. As such, a range of transfusion-sparing strategies have been developed for these individuals and others who refuse standard blood transfusion. Techniques that maintain blood in continuous circuit with the patient are often acceptable and have been used in operative settings, including cell salvage and acute normovolemic hemodilution. By contrast, blood that has been collected and stored for later use has traditionally been considered unacceptable, which has limited the use of both allogeneic and autologous blood products. A March 2026 update by the governing body of Jehovah’s Witnesses, The Watch Tower Bible and Tract Society of Pennsylvania, moves decisions about the storage and reinfusion of a patient’s own blood into the realm of individual conscience, which may expand the use of autologous transfusion. Given this change, requests for autologous transfusion from JW patients may increase. In this letter, we offer practical guidance on balancing respect for patient autonomy and religious belief with the medical and logistical challenges of autologous blood transfusion.

To the Editor,

Refusal of blood transfusion presents a complex challenge in the management of anemic, bleeding, or perioperative patients. Reasons for refusal include concerns about infectious risk, concerns regarding donor vaccination status, and cultural or religious beliefs.^1–3^ Among the most recognized patient populations declining blood products are Jehovah’s Witnesses (JW), who have historically refused allogeneic blood products on religious grounds.^3^ For adult patients with decision-making capacity, this refusal is generally respected as an expression of patient autonomy. Consequently, many strategies have been developed to support patients who decline standard transfusion therapy (ie, blood products from anonymous, nonremunerated, volunteer donors).

These strategies include optimization of erythropoiesis via medication or nutritional supplementation, autologous blood conservation techniques (eg, cell salvage or acute normovolemic hemodilution), and directed or autologous donation.^3^ Historically, for most JW patients, the available options have largely been limited to measures that preserve continuity with the patient’s circulation. Recent shifts in the community religious guidance, however, may broaden consideration of autologous blood use in these patients.

The organization of legal leaders of the congregation of Jehovah’s Witness also known as The Watch Tower Bible and Tract Society has long influenced how members of the JW faith interpret medical use of blood.^4^ Although the original religious prohibition concerned the consumption of blood, this was later extended to transfusion. Over time, JW have generally distinguished between blood that remains in continuous circuit with the patient and blood that is removed and stored. As a result, techniques such as continuous cell salvage and acute normovolemic hemodilution have often been considered matters of individual conscience, whereas preoperative autologous donation has traditionally been declined because the blood is stored outside the body and is no longer in continuity with the circulation.^5^

A March 2026 update appears to broaden this framework by placing the use of a patient’s own blood, including blood that is removed, stored, and later given back, within the domain of personal conscience.^6^ Whether this guidance translates into increased autologous donation will depend on its dissemination among individual patients and congregations, and uptake is likely to vary by region, care setting, and individual conscience rather than following uniformly from the update. Where it is adopted, requests for autologous donation may be expected to increase. Such a development would offer an additional tool for clinicians seeking to provide care that is both clinically appropriate and consistent with patient beliefs, though there are challenges that must be considered.

FDA guidance states that autologous donations should require that a physician order be provided to the blood donation center; the patient must be of reasonable health and have a hemoglobin greater than 11.0 g/dL.^7^ In practice, autologous donation is most feasible for relatively healthy patients with preserved erythropoietic reserves. It is unlikely to be a meaningful option for JW patients who present with symptomatic anemia, ongoing bleeding, or acute transfusion needs in either the outpatient or inpatient setting.

Autologous donation may be of use for JW autologous stem cell transplant (ASCT) recipients. “Bloodless” stem cell transplants have been performed in JW patients,^8^ typically meaning no blood transfusion during the transplant itself. The updated guidance now permits autologous red cells, which could be collected before transplantation for use in the pre-engraftment period. ASCT recipients typically require 1-2 units of red cells during this period, and a single autologous donation may suffice for many patients with modest needs.^8^ However, there are several constraints. Patients undergoing ASCT are often anemic due to chemotherapy, which may render them ineligible to donate. Refrigerated red cells have expiration dates up to 42 days, so units collected before chemotherapy, when the patient is most fit to donate, would expire by the time of stem cell transplant. Red cells can be frozen, often up to 10 years and later thawed, deglycerolized, and transfused,^9^ but blood donor centers generally restrict frozen storage to rare antigen units either for autologous or allogeneic use. At this time, frozen red cells are not typically utilized for standard autologous units.

Even in surgical patients where autologous donation has been used previously, the logistics are challenging. Because blood donor centers typically restrict frozen storage to select cases, donation must occur at most 42 days prior to surgery. Patients may donate a unit up to twice weekly prior to surgery.^10^ Donation itself lowers the patient’s baseline hemoglobin, and recovery before surgery may be incomplete. Thus, although autologous donation may reduce exposure to allogeneic blood, it may also contribute to preoperative anemia if not carefully timed and supported. Prior work has suggested that preoperative autologous donation may, in some settings, be associated with greater perioperative transfusion use, likely reflecting both patient selection and the physiological impact of donation.^11^

These concerns are particularly relevant because the total number of units that can be collected is often limited, commonly to 2 to 3 units.^10^ This may be adequate for selected procedures with modest anticipated blood loss and patients with robust post-donation hemoglobin recovery, but is unlikely to suffice in cases of major hemorrhage. Accordingly, preoperative autologous donation should be viewed as a selective adjunct rather than a comprehensive solution.

If JW patients begin requesting autologous donation more frequently under this updated guidance, clinicians and transfusion services should approach it thoughtfully with a regimented approach (Table 1). Baseline hemoglobin should be assessed, iron, vitamin B12, and folate deficiency should be identified and treated, and erythropoietic support such as intravenous iron or erythropoiesis-stimulating agents may be appropriate in selected cases. Ongoing monitoring during the donation period is essential to avoid worsening preoperative anemia. Clinicians should also recognize that this updated guidance may prompt some patients to reconsider previously documented preferences, and current wishes should be confirmed directly rather than inferred from prior refusals, particularly when decision-making capacity may later be compromised.

**Table 1.** Management of Pre-Operative Blood Refusal Patients Undergoing Autologous Donation

Time Until Date of Surgery	Actions Checklist
Weeks 7-8	Assess patient’s beliefs regarding blood products and transfusion Determine unacceptable blood products (platelets & plasma vs red cells or both) Determine acceptability of blood conservation strategies (eg, cell salvage) and blood alternatives (factor concentrates, albumin, etc.) Document refusal of blood consent and acceptance of any conservation strategies and/or blood alternatives Consult transfusion medicine Obtain labs such as CBC, iron studies, vitamin B12 and folate Supplement iron, vitamin B12, and folate as indicated by lab results
Weeks 6-7	Obtain pre-donation CBC prior to autologous collection for baseline hemoglobin assessment (hemoglobin must be > 11 g/dL) Prescribe autologous red cell donation at local blood donor center
Weeks 2-5	Collect red cells up to twice per week if donor is deemed suitable by the blood donor center policy Monitor hemoglobin weekly Provide IV Iron supplementation throughout entire collection period Cease collection if hemoglobin <11 g/dL
Week 1	Assess for anemia and continue to provide IV Iron supplementation if indicated Confirm number of autologous units within the blood bank
Day of Surgery	Confirm refusal of allogeneic blood and accepted conservation strategies/blood alternatives Utilize Bloodless surgery and anesthesia techniques to minimize bleeding Utilize cell salvage if accepted for intra-operative and immediate post-operative transfusion as an adjunct to donated autologous units in the setting of bleeding Limit lab draws post-operatively to avoid iatrogenic blood loss Post-operative iron supplementation if there is severe anemia

Overall, the recent update may expand the range of blood management options available to some JW patients by allowing consideration of autologous donation as another option for care concordant with their faith. However, its applicability will remain limited by patient health, baseline hemoglobin, procedural timing, and the modest number of units that can typically be collected. For carefully selected preoperative patients, autologous donation may be a reasonable option within a broader blood conservation plan. For many others, especially those with existing anemia or acute transfusion needs, alternative non-transfusion strategies will remain essential.

## AUTHOR CONTRIBUTIONS - CRediT

Conceptualization: Raeshun T. Glover. Writing – original draft: Raeshun T. Glover. Writing – review and editing: Raeshun T. Glover, Brian D. Adkins, Garrett S. Booth, Bipin N. Savani, Jeremy W. Jacobs. All authors approved the final version.

## CONFLICT OF INTEREST STATEMENT

Dr. Jacobs is a member of the Clinical Transfusion Medicine Committee (CTMC) for the Association for the Advancement of Blood and Biotherapies (AABB). The views expressed herein do not necessarily reflect those of the CTMC or AABB.

All other authors declare no competing interest. No external funding or support was received for this work.

## ETHICS APPROVAL STATEMENT

Ethics approval statement is not applicable.

## PATIENT CONSENT STATEMENT

Patient data were not used; therefore, consent was not required.

## Data Availability

Data sharing is not applicable to this article as no datasets were generated or analyzed during the current study.
